# Supplementary material for: LncRNA MIR4435-2HG mediates cisplatin resistance in HCT116 cells by regulating Nrf2 and HO-1
Source: PLoS One. 2020 Nov 24;15(11):e0223035. doi: 10.1371/journal.pone.0223035 (PMC7685444; doi:10.1371/journal.pone.0223035)
Supplement: Attachment — Submitted filename: Minor revisions.docx [file pone.0223035.s001.docx]

Minor revisions

Under *Cell culture and reagents*:

5th line: 2.5x105 change into 2.5x10^5^

9th line: CO2 change into CO_2_

*Cell proliferation assays:*

6th line: CO2 change into CO_2_

References should be homogenous in Vancouver style: e.g. ref. 1: page range should be written as 370-98 etc.
