# Supplementary material for: LncRNA MIR4435-2HG mediates cisplatin resistance in HCT116 cells by regulating Nrf2 and HO-1
Source: PLoS One. 2020 Nov 24;15(11):e0223035. doi: 10.1371/journal.pone.0223035 (PMC7685444; doi:10.1371/journal.pone.0223035)
Supplement: Attachment — Submitted filename: Response to Reviewers.docx [file pone.0223035.s002.docx]

Subject: Manuscript PONE-D-19-25514, entitled "LncRNA MIR4435-2HG mediates cisplatin resistance in HCT116 cells by regulating Nrf2 and HO-1".

We deeply appreciate your consideration of our manuscript for publication in your journal.

We have revised the above manuscript according to the journal editor and reviewer's comments.

**1. We noticed you have some minor occurrence of overlapping text with the following previous publication(s), which needs to be addressed:**

[**https://doi.org/10.1111/jgh.13069**](https://doi.org/10.1111/jgh.13069)

[**https://doi.org/10.1097/PGP.0000000000000079**](https://doi.org/10.1097/PGP.0000000000000079)

[**https://doi.org/10.7717/peerj.6683**](https://doi.org/10.7717/peerj.6683)

[**https://doi.org/10.2147/DDDT.S106412**](https://doi.org/10.2147/DDDT.S106412)

**In your revision ensure you cite all your sources (including your own works), and quote or rephrase any duplicated text outside the Methods section. Further consideration is dependent on these concerns being addressed.**

Thanks for your suggestion and hard work. We have revised this section in our MS and marked in

blue as your suggestion. And we have also revised some other small sections in our MS and

marked in red as your suggestion.

**2. Please note that all PLOS journals ask authors to adhere to our policies for sharing of data and materials: https://journals.plos.org/plosone/s/data-availability. According to PLOS ONE’s Data Availability policy, we require that the minimal dataset underlying results reported in the submission must be made immediately and freely available at the time of publication. As such, please remove any instances of 'unpublished data' or 'data not shown' in your manuscript and replace these with either the relevant data (in the form of additional figures, tables or descriptive text, as appropriate), a citation to where the data can be found, or remove altogether any statements supported by data not presented in the manuscript. Particularly, either include as a Supporting file the RNA-seq data you mention at the beginning of your Results section and add the corresponding methodology in the Methods section, or consider removing the sentence altogether.**

Thanks for your suggestion and hard work. We agree and stick to the policies of PLOS journals for sharing our data and material at the time of publication.

**3. PLOS requires an ORCID ID for the corresponding author in Editorial Manager on papers submitted after December 6th, 2016. Please ensure that you have an ORCID iD and that it is validated in Editorial Manager. To do this, go to ‘Update my Information’ (in the upper left-hand corner of the main menu), and click on the Fetch/Validate link next to the ORCID field. This will take you to the ORCID site and allow you to create a new iD or authenticate a pre-existing iD in Editorial Manager. Please see the following video for instructions on linking an ORCID iD to your Editorial Manager account:** [**https://www.youtube.com/watch?v=_xcclfuvtxQ**](https://www.youtube.com/watch?v=_xcclfuvtxQ)

Thanks for your suggestion and hard work. The ORCID iD related to corresponding author (Xiaoming Zhong) is 0000-0002-3535-6678.

**4. Under Cell culture and reagents: 5th line: 2.5x105 change into 2.5x10^5^and 9th line: CO2 change into CO_2；_Cell proliferation assays: 6th line: CO2 change into CO_2._**

Thanks for your suggestion and hard work. We have revised this section in our MS and marked in

red as your suggestion.

5. **References should be homogenous in Vancouver style: e.g. ref. 1: page range should be written as 370-98 etc.**

Thanks for your suggestion and hard work. We have revised this section in our MS according to previous article in the journal of PloS One [1] and marked in yellow as your suggestion.

[1] Takenaga K, Akimoto M, Koshikawa N, Nagase H. Cancer cell-derived interleukin-33 decoy receptor sST2 enhances orthotopic tumor growth in a murine pancreatic cancer model. **PLoS One.** **2020**; 15: e0232230. https://doi.org/10.1371/journal.pone.0232230 PMID: 32340025

**Comments to the Author**

**1. Is the manuscript technically sound, and do the data support the conclusions?**

**The manuscript must describe a technically sound piece of scientific research with data that supports the conclusions. Experiments must have been conducted rigorously, with appropriate controls, replication, and sample sizes. The conclusions must be drawn appropriately based on the data presented.**

**Reviewer #1: Yes**

Thanks for your comment and hard work.

**2. Has the statistical analysis been performed appropriately and rigorously?**

**Reviewer #1: Yes**

Thanks for your comment and hard work.

**3. Have the authors made all data underlying the findings in their manuscript fully available?**

**The PLOS Data policy requires authors to make all data underlying the findings described in their manuscript fully available without restriction, with rare exception (please refer to the Data Availability Statement in the manuscript PDF file). The data should be provided as part of the manuscript or its supporting information, or deposited to a public repository. For example, in addition to summary statistics, the data points behind means, medians and variance measures should be available. If there are restrictions on publicly sharing data—e.g. participant privacy or use of data from a third party—those must be specified.**

**Reviewer #1: Yes**

Thanks for your comment and hard work.

4. Is the manuscript presented in an intelligible fashion and written in standard English?

PLOS ONE does not copyedit accepted manuscripts, so the language in submitted articles must be clear, correct, and unambiguous. Any typographical or grammatical errors should be corrected at revision, so please note any specific errors here.

Reviewer #1: Yes

Thanks for your comment and hard work.

**5. Review Comments to the Author:**

**Please use the space provided to explain your answers to the questions above. You may also include additional comments for the author, including concerns about dual publication, research ethics, or publication ethics. (Please upload your review as an attachment if it exceeds 20,000 characters)**

**Reviewer#1: The Cohen's d factors are large enough so the results are clear and unambiguous. The results may provide the useful tool how to circumvent the resistance to cisplatin beside the determination of polimorfisms of proteins involved in transport of cisplatin.**

Thanks for your comment and hard work.

**6. PLOS authors have the option to publish the peer review history of their article (what does this mean?). If published, this will include your full peer review and any attached files.**

**If you choose “no”, your identity will remain anonymous but your review may still be made public.**

**Do you want your identity to be public for this peer review? For information about this choice, including consent withdrawal, please see our Privacy Policy.**

**Reviewer #1: Yes: Tomaž Makovec**

Thank you for your option and hard work.
